# 云南省肿瘤医院2005年-2014年肺癌流行病学特征变化趋势分析

**DOI:** 10.3779/j.issn.1009-3419.2020.03.05

**Published:** 2020-03-20

**Authors:** 艳苹 林, 强 张, 彦霓 陆, 云超 黄, 洁 马, 永春 周

**Affiliations:** 650118 昆明，云南省肿瘤医院/昆明医科大学第三附属医院/云南省癌症中心 Department of Yunnan Cancer Center, Yunnan Cancer Hospital/The Third Affiliated Hospital of Kunming Medical University/Yunnan Cancer Center, Kunming 650118, China

**Keywords:** 肺肿瘤, 临床流行病学, 云南, Lung neoplasmas, Clinical epidemiology, Yunnan

## Abstract

**背景与目的:**

云南省是全国乃至世界肺癌的高发区，其发病率和死亡率仍处于不断上升趋势。随着生活方式及环境因素的改变，肺癌的临床流行病学特征在发生改变。但近10年来云南省肺癌临床特征的变化趋势未见报道，需进一步研究。本研究旨在探讨2005年-2014年云南地区肺癌临床特征及其变化趋势，为本地区的肺癌防治提供理论依据。

**方法:**

采用回顾性调查研究方法，通过简单随机抽样抽取本院2005年-2014年的肺癌住院患者，应用统一、规范的调查问卷提取患者的社会人口学及临床病理特征，并进行数据统计分析。

**结果:**

共纳入1, 000例肺癌患者，平均年龄（58.1±10.1）岁，男女比例3.08/1.00。男性患者比例从2005年的75.0%下降至2014年的66.0%，而女性患者从25.0%增加到34.0%（*P*=0.007）。年龄≥60岁患者比例从2005年的30.0%增加到2014年的39.0%，60岁以下患者占比有所下降，但无统计学差异（*P*=0.532）。受教育水平较低（小学或初中）患者的比例从36.0%增加到66.0%（*P* < 0.001）。吸烟患者占比从71.0%下降至47.0%，不吸烟人群从29.0%上升至52.0%（*P*=0.003）。晚期肺癌（Ⅲb期-Ⅳ期）患者从20.0%上升至54.0%，而Ⅱ期-Ⅲa期比例从62.0%下降至24.0%（*P*=0.002）。腺癌占比从36.0%增加至61.0%，而鳞状细胞癌从32.0%下降至27.0%（*P* < 0.001）。胸部X射线应用从91.0%下降到58.0%（*P* < 0.001），而胸部计算机断层扫描（computed tomography, CT）使用率从46.0%增加到89.0%（*P* < 0.001）。头部磁共振成像（magnetic resonance imaging, MRI）使用率由1.0%上升至15.0%（*P* < 0.001）。骨扫描从35.0%上升至78.0%（*P* < 0.001）。高成本的正电子发射计算机断层显像（positron emission tomography-CT, PET-CT）检查技术从0.0%显著上升至17.0%。化疗（*P*=0.67）和手术（*P*=0.78）是最常用的治疗方式，且在过去10年间治疗方式不变。

**结论:**

女性患者比例增加、临床分期较晚、病理类型转变等是目前云南地区肺癌防治面临的重要趋势。尽管社会人口学及临床病理特征发生较大变化，但主要治疗方式的选择仍未发生改变，需进一步研究。

## Abstract (en)

**Background and Objective:**

Yunnan is a country with a high incidence of lung cancer in China and all over the world, and its morbidity and mortality are still rising. With changes in lifestyle and environment, the clinical epidemiological characteristics of lung cancer are converting. However, the trend of clinical characteristics of lung cancer in Yunnan has not been reported in the past 10 years, and we should start further research. The aim of this study was to explore the clinical characteristics and changes of lung cancer in Yunnan from 2005 to 2014, and to provide a theoretical basis for lung cancer prevention and treatment in this region.

**Methods:**

A retrospective survey was used to extract the cases of lung cancer patients who were treated in our hospital from 2005 to 2014 by simple random sampling. The sociodemographic and clinicopathological characteristics of the patients were extracted by using a unified and standardized questionnaire. And the statistical analysis of the data was performed.

**Results:**

A total of 1, 000 patients with lung cancer were enrolled, with an average age of (58.1±10.1) years, and the ratio of male to female was 3.08/1.00. The proportion of male patients decreased from 75.0% in 2005 to 66.0% in 2014, while female patients increased from 25.0% to 34.0% (*P*=0.007). The proportion of patients aged ≥60 years increased from 30.0% in 2005 to 39.0% in 2014, and the proportion of patients under 60 years of age decreased, but there was no statistical difference (*P*=0.532). The proportion of patients with lower levels of education (primary or junior high school) increased from 36.0% to 66.0% (*P* < 0.001). The proportion of smokers decreased from 71.0% to 47.0%, and the number of non-smokers increased from 29.0% to 52.0% (*P*=0.003). The patients with advanced lung cancer (Ⅲb-Ⅳ) increased from 20.0% to 54.0%, while the proportion of stage Ⅱ-Ⅲa decreased from 62.0% to 24.0% (*P*=0.002). The proportion of adenocarcinoma increased from 36.0% to 61.0%, while squamous cell carcinoma decreased from 32.0% to 27.0% (*P* < 0.001). Chest X-ray applications decreased from 91.0% to 58.0% (*P* < 0.001), while chest computed tomography (CT) usage increased from 46.0% to 89.0% (*P* < 0.001). Head magnetic resonance imaging (MRI) usage increased from 1.0% to 15.0% (*P* < 0.001). The bone scan increased from 35.0% to 78.0% (*P* < 0.001). The positron emission tomography-CT (PET-CT) inspection technique increased significantly from 0.0% to 17.0%. Chemotherapy (*P*=0.67) and surgery (*P*=0.78) were the most common treatments and the treatments were unchanged over the past 10 years.

**Conclusion:**

The proportion of female patients increased, the clinical stage was late, and the pathological type transformation was a major challenge in the prevention and treatment of lung cancer in Yunnan. Despite major changes in sociodemographic and clinicopathological features, the choice of primary treatment modalities has not changed, and further research is needed.

肺癌是癌症发病和死亡的首要原因。世界估计每年有180万新发病例和160万死亡病例，其中中国新增病例占35.8%，死亡病例占37.6%^[1]^。以人群为基础的肿瘤登记数据^[2]^显示，肺癌是中国最常见的癌症，在过去的10年中，女性的发病率不断增加，中国面临着严重的肺癌疾病负担。

肺癌预后不良，在中国5年生存率仅为16.1%，不同人口学特征和临床病例特征将影响患者的预后^[3]^。例如，在美国局限期肺癌的5年生存率为50.7%，而晚期肺癌仅为4.3%^[4]^。因此，掌握肺癌的社会人口学分布特征和临床病理特征是肺癌预防决策及其预后判断的依据。据国家癌症中心报道^[5]^2005年-2014年间，中国肺癌的流行病学面临着居高不下的吸烟率、女性肺癌发病率不断上升、临床分期较晚、主要病理类型的转变以及医疗费用不断增长的现状。这是中国目前肺癌防治的挑战，同时也为未来的肺癌防控措施指明了方向。

众所周知，云南省肺癌发病率较高，尤其宣威地区是全国乃至世界肺癌的高发区。2014年云南省肿瘤登记地区恶性肿瘤发病与死亡分析^[6]^显示，肺癌是云南省恶性肿瘤发病和死亡的首要原因，居男性恶性肿瘤发病和死亡第1位，女性恶性肿瘤发病第2位、死亡第1位。农村地区肺癌的发病率和死亡率高于城市地区。云南的肺癌表现为点状区域性分布，主要集中在宣威和个旧两个地区。

但是，目前尚未报道近10年来云南省肺癌临床特征的变化趋势，其特征变化是否与全国及其他地区有所差异，需进一步研究。本研究为国家癌症中心牵头的全国多中心城市癌症早诊早治项目中卫生经济学调查研究，其研究方案按照《2015-2016年度全国技术方案与现场执行手册卫生经济学评价部分》，相关问卷及方案由国家癌症中心提供。采用回顾性的临床流行病学研究旨在调查云南省肺癌临床特征的现状及其变化，包括社会人口学和临床病理学特征、诊断方法和治疗方案，现将云南省肿瘤医院单中心的相关研究结果报道如下，以期为云南省肺癌防治策略提供参考依据。

## 1 材料和方法

### 1.1 研究对象

纳入标准：①确诊为原发性肺癌的既往患者（首诊日期在2005年1月1日-2014年12月31日间）；②曾经在云南省肿瘤医院住院治疗，且接受了主要治疗（仅在该院接受诊断或术后随访的患者应予以排除）；③临床资料信息完整，包括临床诊断[临床分期或者肿瘤原发灶-淋巴结-转移（tumor-node-metastasis, TNM）分期信息二者至少必备其一]、治疗方案和病理诊断信息。

### 1.2 抽样方法

本研究为全国多中心研究，1年内去掉春节前后的1月份和2月份，在其余3月份-12月份内进行如下抽样：将各研究医院按照项目编号排列后采用随机函数UNIFORM（*n*）产生相应的随机数，对该随机数由小到大排序，以秩次号作为选取各医院2005年需要收集病例的月份的依据，秩次号最小（编号1）的医院，2005年需收集该院3月份所有的病案资料，2006年需收集该院4月份所有病案资料，依次类推。我院随机编号为2，2005年收集4月份病例资料，2006年收集5月份病例资料，以此类推。若起始月份符合入组病例不足100例，则继续往后地毯式推进，不挑不跳，直到收满当年100例为止。

### 1.3 调查问卷

由国家癌症中心肿瘤流行病学、病理学、诊断学、放射学、胸外科、肿瘤内科、放射肿瘤学等组成的多学科专家小组设计了一份11页的肺癌诊疗信息调查问卷。这份长达11页的调查问卷包括：社会人口统计学和肺癌危险因素、临床症状和体征、并发症和临床特征、临床分期和病理类型、医学影像学、纤维支气管镜、实验室检查、病理学等诊断方法利用率以及不同的治疗方式。

### 1.4 数据收集和质量控制

所有数据均通过医疗记录系统批量导出或手动查询。为了提高数据质量，问卷调查员具有两年以上肺癌临床经验的医生或研究生，或来自肿瘤防控部门具有临床医学或公共卫生教育背景的工作人员。在数据收集过程中，调查员首先根据医院的记录排查是否符合研究入组条件，然后提取完整的调查信息。为了实现所有数据的一致性，问卷均采用双重录入EpiData数据库，然后统一使用SAS软件进行缺失值确认和逻辑检查。

### 1.5 统计分析

计数资料以频率表示，并使用卡方检验或*Fisher*确切概率法，包括性别、婚姻、职业、教育水平、医疗保险类型、医疗保险所在地、体质指数、吸烟史、饮酒史、慢性呼吸系统病史、临床分期、病理类型、诊断技术利用和治疗方案。计量资料使用*t*检验进行分析。2005年-2014年间各变量的变化趋势采用趋势卡方检验。所有数据使用SPSS 19.0统计软件建立数据库及统计分析，*P* < 0.05认为有统计学差异。

## 2 结果

共有1, 064例肺癌患者最初入组，经过数据核查后64例信息不全，共计1, 000例原发性肺癌患者病例进行最终分析，纳入率为94.0%。

### 2.1 整体社会人口学和临床病理特征

在1, 000例肺癌患者中，2005年-2009年诊断病例493例，2010年-2014年诊断病例507例。所有纳入病例诊断时的平均年龄为（58.1±10.1）岁，男女比例为3.08/1.00（755/245）。正在吸烟者487例（48.7%），曾经吸烟者78例（7.8%）。对于临床分期，Ⅰ期、Ⅱ期-Ⅲa期和Ⅲb期-Ⅳ期患者分别为209例（20.9%）、392例（39.2%）和399例（39.9%）。病理类型腺癌最多，占49.4%；其次是鳞状细胞癌，占33.1%；小细胞癌占10.5%，其他类型占7.0%。除婚姻状况、体质指数、饮酒史和慢性呼吸系统疾病史外，近5年（2010年-2014年）其他社会人口学和临床病理特征较2005年-2009年有显著统计学差异（*P* < 0.05）。具体数据详见表 1。

**1:** 1, 000例肺癌患者的社会人口学和临床病理特征[*n*（%）] Sociodemographic and clinicopathological characteristics of 1, 000 lung cancer patients [*n* (%)]

Characteristic	Total (*n*=1, 000)	2005-2009 (*n*=493）	2010-2014 (*n*=507）	*P*
Gender				0.06
Male	755 (75.5)	385 (78.1)	370 (73.0)	
Female	245 (24.5)	108 (21.9)	137 (27.0)	
Age at diagnosis (Mean±SD, yr)	58.1±10.1	58.4±10.3	57.8±9.8	< 0.001
Age at diagnosis (yr)				0.911
< 45	164 (16.4)	82 (16.6)	82 (16.2)	
45-59	450 (45.0)	224 (45.4)	226 (44.6)	
≥60	386 (38.6)	187 (38.0)	199 (39.2)	
Marriage status				0.132
Married	987 (98.7)	486 (98.6)	501 (98.8)	
Unmarried	8 (0.8)	5 (1.0)	3 (0.6)	
Unknown	5 (0.5)	2 (0.4)	3 (0.6)	
Occupation				< 0.001
Farmer	449 (44.9)	174 (35.3)	275 (54.2)	
Enterprise employee	175 (17.5)	102 (20.7)	73 (14.4)	
Self-employed	12 (1.2)	7 (1.4)	5 (1.0)	
Unemployed	48 (4.8)	36 (7.3)	12 (2.4)	
Public sector employee	134 (13.4)	87 (17.6)	47 (9.3)	
Retirement	114 (11.4)	54 (11.0)	60 (11.8)	
Other	43 (4.3)	17 (3.4)	26 (5.1)	
Unknown	25 (2.5)	16 (3.2)	9 (1.8)	
Education level				< 0.001
Primary school or below	416 (41.6)	190 (38.5)	226 (44.6)	
High school or above	387 (38.7)	258 (52.3)	129 (25.4)	
Unknown	197 (19.7)	45 (9.2)	152 (30.0)	
Health insurance status				< 0.001
Employee medical insurance	220 (22.0)	116 (23.5)	104 (20.5)	
Resident medical insurance	378 (37.8)	238 (48.3)	140 (27.6)	
Self pay	218 (21.8)	112 (22.7)	106 (20.9)	
Other	156 (15.6)	10 (2.0)	146 (28.8)	
Unknown	28 (2.8)	17 (3.5)	11 (2.2)	
Health insurance location				0.001
Local	186 (18.6)	95 (19.30)	91 (17.9)	
Non-local	775 (77.5)	368 (74.6)	407 (80.3)	
Unknown	39 (3.9)	30 (6.1)	9 (1.8)	
BMI (kg/m^2^)				0.33
< 18.5	85 (8.5)	49 (9.9)	36 (7.1)	
18.5-23.9	678 (67.8)	335 (68.0)	343 (67.6)	
24.0-27.9	203 (20.3)	93 (18.9)	110 (21.7)	
≥28.0	34 (3.4)	16 (3.2)	18 (3.6)	
Smoking status				< 0.001
Current smoker	487 (48.7)	244 (49.5)	243 (47.9)	
Former smoker	78 (7.8)	56 (11.4)	22 (4.3)	
Never smoker	423 (42.3)	186 (37.7)	237 (46.8)	
Unknown	12 (1.2)	7 (1.4)	5 (1.0)	
Alcohol consumption status				0.327
Yes	320 (32.0)	166 (33.7)	154 (30.4)	
No	661 (66.1)	320 (64.9)	341 (67.3)	
Unknown	19 (1.9)	7 (1.4)	12 (2.3)	
History of chronic respiratory disease				0.453
Yes	38 (3.8)	21 (4.3)	17 (3.4)	
No	962 (96.2)	472 (95.7)	490 (96.6)	
Clinical stage				< 0.001
Ⅰ	209 (20.9)	103 (20.9)	106 (20.9)	
Ⅱa	101 (10.1)	52 (10.5)	49 (9.7)	
Ⅱb	91 (9.1)	61 (12.4)	30 (5.9)	
Ⅲa	200 (20.0)	114 (23.1)	86 (17.0)	
Ⅲb	117 (11.7)	60 (12.2)	57 (11.2)	
IV	282 (28.2)	103 (20.9)	179 (35.3)	
Pathological type				< 0.001
Adenocarcinoma	494 (49.4)	202 (41.0)	292 (57.6)	
Squamous cell carcinoma	331 (33.1)	188 (38.1)	143 (28.2)	
Small cell carcinoma	105 (10.5)	53 (10.8)	52 (10.3)	
Other	70 (7.0)	50 (10.1)	20 (3.9)	

### 2.2 社会人口学和临床病理特征的变化趋势

2005年-2014年，10年间男性肺癌患者比例下降，从2005年的75.0%到2014年的66.0%，而女性患者比例则从25.0%上升至34.0%（*P*=0.007）。年龄≥60岁患者比例从2005年的30.0%增长到2014年的39.0%，60岁以下患者占比有所下降，但无统计学差异（*P*=0.532）。受教育水平较低（小学或初中）患者的比例从2005年的36.0%增加到2014年的66.0%（*P* < 0.001）。值得注意的是，正在吸烟者从54.0%降至44.0%，曾经吸烟者比例从17.0%降至3.0%，不吸烟人群从29.0%上升至52.0%（*P*=0.003）。晚期肺癌（Ⅲb期-Ⅳ期）患者从20.0%上升至54.0%，而Ⅱ期-Ⅲa期患者比例从62.0%下降至24.0%（*P*=0.002）。关于病理类型，腺癌占比从36.0%增加至61.0%，而鳞状细胞癌从32.0%下降至27.0%（*P* < 0.001）。详见图 1-图 6。

**1:**
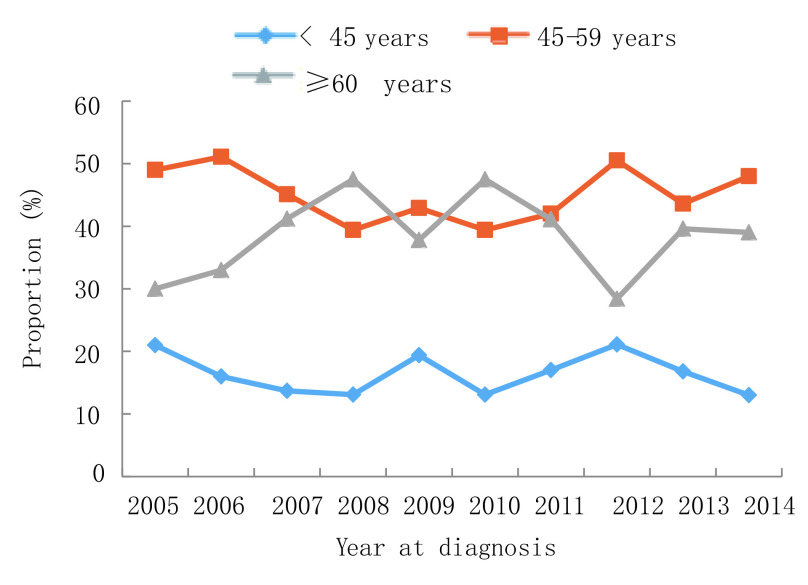
年龄变化趋势 Trend of age

**2:**
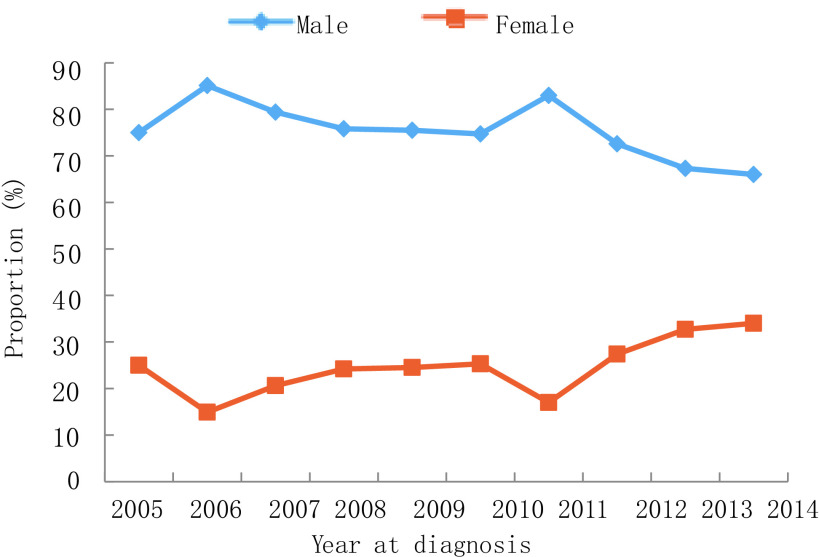
性别变化趋势 Trend of gender

**3:**
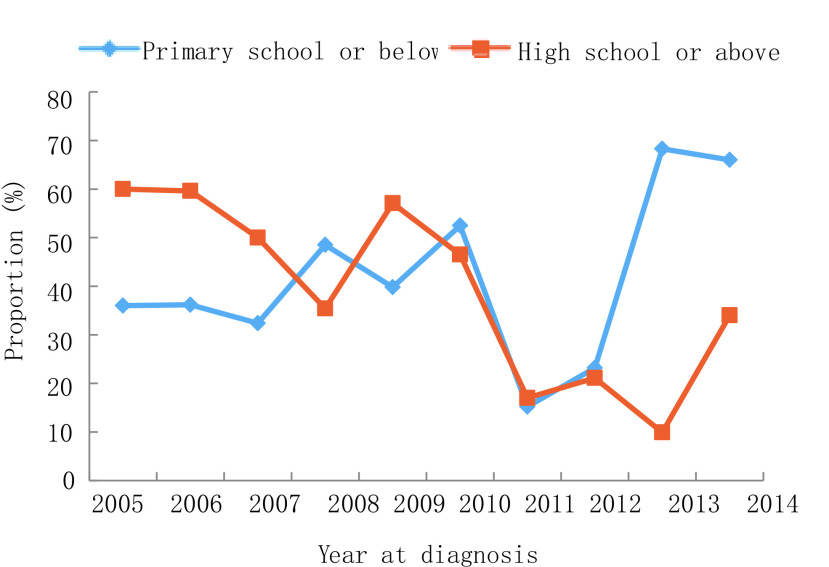
受教育水平变化趋势 Trend of education level

**4:**
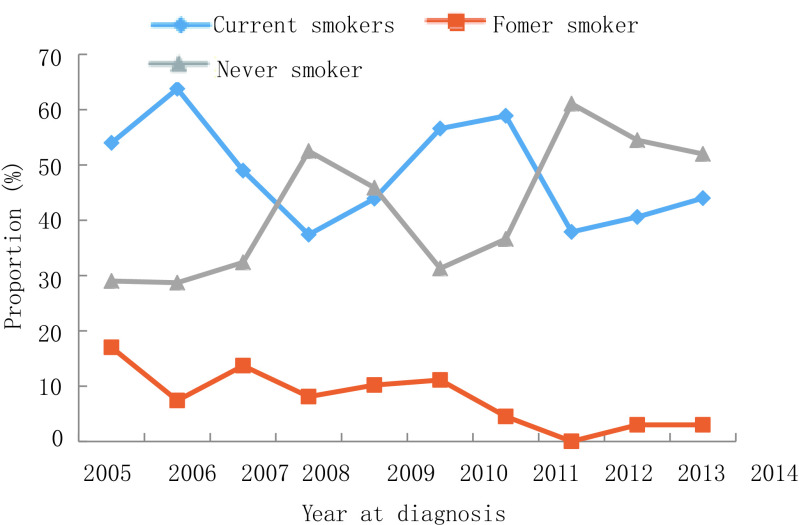
吸烟史变化趋势 Trend of smoking status

**5:**
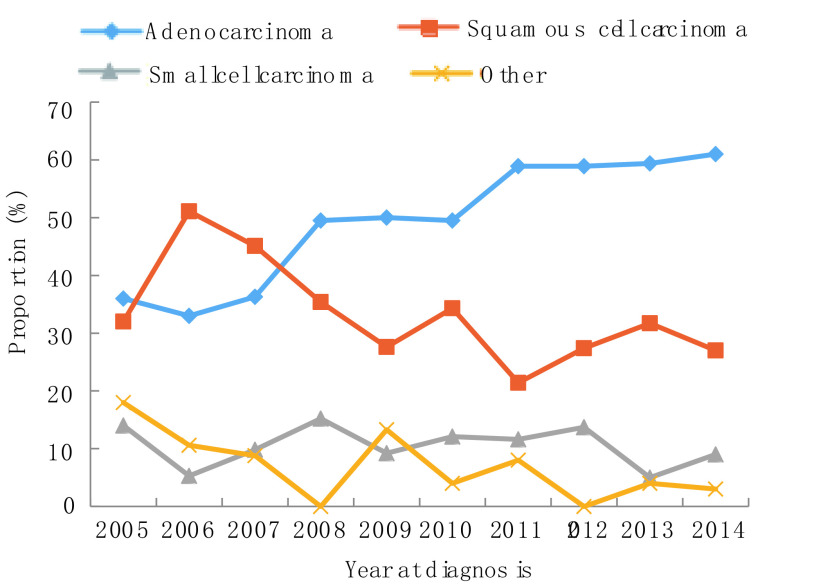
病理类型变化趋势 Trend of pathological subtype

**6:**
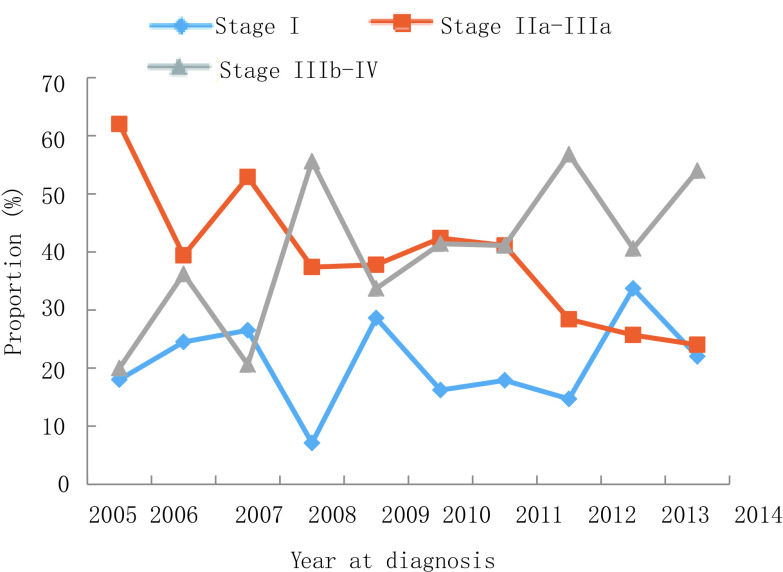
临床分期变化趋势 Trend of clinical stage

### 2.3 整体医疗服务利用情况

关于肺癌诊断技术，胸部计算机断层扫描（computed tomography, CT）是使用最广泛的诊断技术之一（730例，73.0%），大多数患者也进行了胸部X射线检查（702例，70.2%）。47.8%的患者使用头部CT，15.5%的患者进行了头部核磁共振成像（magnetic resonance imaging, MRI）。只有58例患者使用正电子发射断层显像/计算机断层扫描（positron emission tomography/CT, PET-CT），占5.8%。支气管镜检查作为一种常用的诊断技术，有78.0%的患者接受了该项检查。75.5%的患者接受了骨扫描。治疗方式主要为手术和化疗，极少部分患者接受放疗和靶向治疗。50.0%的患者接受手术治疗，52.2%的患者接受化疗，0.9%的患者接受放疗，0.2%的患者接受靶向治疗。

### 2.4 医疗服务利用情况变化趋势

与2005年-2009年相比，2010年-2014年接受胸部CT（*P* < 0.001）、头部MRI（*P* < 0.001）、骨扫描（*P* < 0.001）和PET-CT（*P* < 0.001）检查的患者逐年增多，而接受胸部X射线（*P* < 0.001）检查的患者逐渐减少。从2005年-2014年，胸部X射线的应用从91.0%下降到58.0%（*P* < 0.001），但胸部CT的使用率从46.0%增加到89.0%（*P* < 0.001）。头部MRI从2005年的1.0%不断增加到2014年的15.0%（*P* < 0.001）。骨扫描的应用从35.0%上升到78.0%。高成本的PET-CT检查技术从0.0%显著上升至17.0%。详见图 7-图 8。而纤支镜、头颅CT检查的应用在10年间无明显差异。在所有治疗方案中，化疗（*P*=0.67）和手术（*P*=0.78）是最常用的治疗方式，且在过去10年间治疗方式占比基本不变。

**7:**
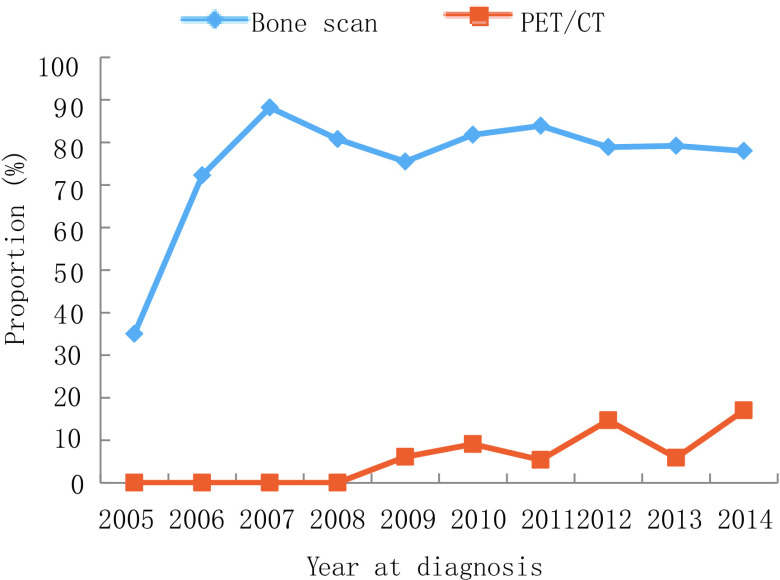
PET/CT和骨扫描检查使用率变化趋势 Trend of other PET/CT and bone scan. PET-CT: positron emission tomography-computed tomography

**8:**
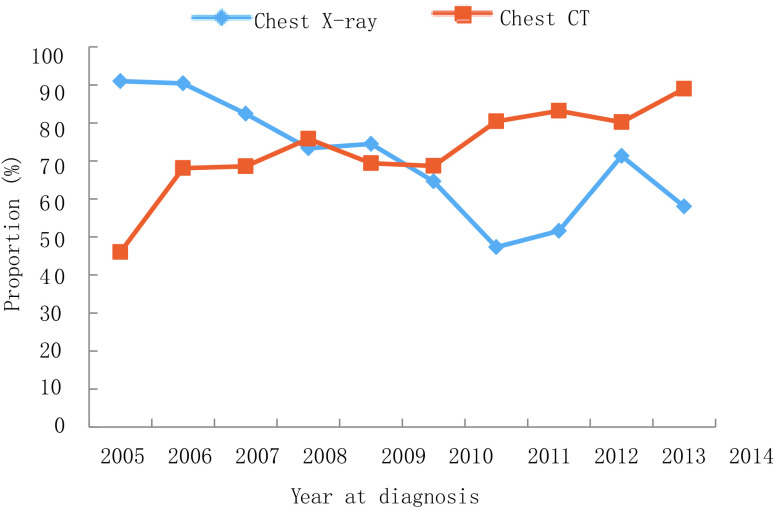
胸部检查方法变化趋势 Trend of chest examination

## 3 讨论

这是首次对近10年来云南省肿瘤医院肺癌的流行病学调查研究，结果显示，2005年-2014年云南地区肺癌患者的社会人口学及临床病理特征有较大变化，如女性患者比例不断上升，临床分期上升和病理类型的变化。诊断技术的使用发生了很大变化，但主要治疗方案保持不变。我们的研究结果为云南地区肺癌的主要防控方向和临床实践提供理论依据。

该研究发现，2010年-2014年诊断时的平均年龄略高于2005年-2009年，这表明老年（年龄≥60岁）患者比例增加。国家癌症中心肿瘤登记处的数据^[7]^显示中国老年患者的发病率是所有人口的6倍。我们研究发现云南地区肺癌诊断时的平均年龄为58.1岁，与全国平均水平接近，但低于美国报告的71岁^[8]^和澳大利亚报道的68岁^[9]^。人口老龄化的持续加速可能会进一步加剧老年人口的肺癌疾病负担^[10]^。性别比例趋势分析显示，男性占比呈下降趋势，女性占比呈上升趋势。国家肿瘤登记处数据^[11]^显示女性的肺癌发病率显著增加而男性则逐渐减少，这与我们的研究结果一致。男性发病率逐年下降的主要原因可能是肺癌患者和一般人群的吸烟率下降，但稳定的女性吸烟率无法解释其逐渐上升患病率趋势^[12, 13]^，二手烟暴露可能是一个主要的危险因素^[14]^。中国女性二手烟的暴露率和不吸烟流行率相当，约71.6%，估计占肺癌病例的24.0%；大多数中国传统女性暴露于室内空气燃烧产物的污染，约占肺癌病例的20.0%^[15]^。其他危险因素包括肺结核、氡污染和室外空气，也可能导致女性肺癌发病率上升^[16]^。

病理类型变化趋势研究发现腺癌比例逐年增加，鳞状细胞癌比例逐渐下降，这与北京^[17, 18]^、四川和美国、欧洲等^[19, 20]^国家报道一致。但在主要病理类型的变化趋势上也有别于全国情况，表现在2005年-2014年的10年间云南地区肺癌的主要病理类型均为腺癌，而中国的主要病理类型是由鳞状细胞癌逐渐转变为腺癌^[17, 18]^。导致病理类型变化的一个关键因素是吸烟率变化，研究^[21]^发现鳞癌患者的吸烟率高于腺癌和小细胞癌。本研究发现Ⅲb期-Ⅳ期患者比例增加，Ⅱ期-Ⅲa期患者比例下降，这与美国2004年-2010年期间^[22]^和英国2008年-2012年期间^[23]^变化趋势一致。本研究中高中及其以上教育水平患者比例持续下降，而小学及以下教育水平患者比例逐年上升。这可能与不同教育水平人群的生活方式和健康意识有关。低剂量CT筛查已被随机对照试验证实为一种减少晚期肺癌患者及降低肺癌死亡率的有效方法^[24]^，但中国自2009年开展农村肺癌筛查和2012年开展城市肺癌筛查以来，晚期肺癌分期占比未下降^[5]^，云南地区与全国同步开展肺癌筛查，肺癌分期构成变化趋势一致。

医疗服务利用分析结果显示，诊断技术取得了实质性的改进，而主要治疗方案随着时间的推移没有明显的变化。辅助检查手段由胸部X射线转向胸部CT，越来越多的患者采用纤维支气管镜检查、骨扫描、PET/CT等。本研究中靶向治疗率较低，可能与本地区*EGFR*基因突变频率、靶向治疗费用较高、经济欠发达以及研究时段内靶向治疗药物尚未进入医保支付范围等有关。此外，放射治疗比率也较低，可能与本地区放射治疗资源有限、同期放化疗患者较少、大多数局部晚期患者进行单纯化疗等因素有关。

本研究仅在云南省肿瘤医院院内进行抽样调查，其研究结果具有一定的代表性（本院收治肿瘤患者约占全省肿瘤患者的70%左右），但也存在一定局限性。回顾性研究可能与相关医疗记录系统的完整性和准确性有关。本研究数据分析缺乏治疗手段等亚组分析，通过描述统计学分析相关结果可能存在潜在的混杂因素。总之，本研究采用回顾性流行病学调查方法，描述了2005年-2014年云南地区大多数肺癌患者的人口社会学和临床病理特征的变化趋势，为云南地区肺癌的防治提供理论依据。

## Author contributions

Huang YC and Zhou YC conceived and designed the study. Lin YP and Zhang Q performed the experiments. Lin YP analyzed the data. Ma J contributed analysis tools. Lin YP, Zhang Q, Lu YN, Huang YC, Ma J and Zhou YC provided critical inputs on design, analysis, and interpretation of the study. All the authors had access to the data. All authors read and approved the final manuscript as submitted.
